# Human Polyomavirus-Associated Cerebral Disorders in the Post-HAART Era

**DOI:** 10.4061/2011/562427

**Published:** 2011-02-22

**Authors:** Filiberto Cedeno-Laurent, Augusto C. Penalva de Oliveira, José E. Vidal, J. Roberto Trujillo

**Affiliations:** ^1^Harvard Medical School, Boston, MA 02215, USA; ^2^Department of Neurology, Institute of Infectious Diseases “Emilio Ribas,” 040088-002, São Paulo, SP, Brazil; ^3^Unit on Clinical Investigation of Human Retroviruses, Campinas University, São Paulo, Brazil; ^4^Department of Infectious Diseases, Institute of Infectious Diseases “Emilio Ribas,” 040088-002, São Paulo, SP, Brazil; ^5^TruBios Research Institute, Johns Hopkins University, MCC, 9605 Medical Center Drive, Suite 105, Rockville, MD 20850, USA

## Abstract

Human polyomavirus JC is the causative agent of a deadly form of sudden onset dementia, progressive multifocal leukocoencephalopathy (PML). PML is highly prevalent in immunodeficient populations, specially those undergoing chemotherapy, immunosuppressive treatments for autoimmune conditions, and HIV-1/AIDS patients. In fact, before the highly active antiretroviral therapy (HAART) regimens became available, PML was a leading cause of death in HIV-1 seropositive individuals. However, patients under HAART show increased survival times with better prognoses. In this report we described the main differences between PML before and after the HAART era; highlighting the new patterns of presentation, the neurotropism of other human polyomaviruses, and the increased prevalence of immune reconstitution inflammatory syndrome (IRIS), as a complication of PML in patients under HAART. Lastly, we propose a revised classification of human poliomavirus-associated cerebral disorders that may reflect more accurately what clinicians encounter in their everyday practice.

Since the introduction of highly active antiretroviral therapy (HAART), morbidity and mortality secondary to opportunistic disorders in patients infected with HIV-1 have significantly decreased [1]. However, the prevalence of neurocognitive impairment has remained steady and still occurs in up to 50% of HIV-1/AIDS patients [2]. Moreover, there is a growing number of reports depicting atypical presentations of previously well-characterized brain disorders [3], as well as emerging pathologies associated with the reactivation of the immune system [4], both of which represent diagnostic and therapeutic challenges to experts in the fields of neurology and infectious diseases.

Human polyomaviruses JC and BK (named after the two initials of the first patients in which the viruses were, respectively, isolated) rarely cause disease, except for patients with an underlying immunocompromise. JC virus (JCV) is the causative agent of a fatal demyelinating disorder termed progressive multifocal leukoencephalopathy (PML) [5]. PML is an unusual source of cognitive, motor, and visual impairments with rapid death in the absence of HAART. This disorder is recognized by multiple foci of demyelination affecting the subcortical white matter, and by pathological findings that include giant astrocytes and olygodendrocytes with enlarged nuclei. Similarly, BK virus (BKV) is known to cause hemorrhagic cystitis and polyomavirus-associated nephropathy (PVAN), especially in kidney transplant recipients and in patients undergoing chemotherapy.

Before HAART became widely available, there was no treatment for polyomavirus-associated disorders, and death from PML occurred as early as 3 months after diagnosis [6]. The one-year survival of patients with PML has substantially increased over the last decade, from 0%–30% before HAART became available to 38%–62% in the post-HAART era [7–10]. Interestingly, while the prognosis and survival of patients with PML have greatly increased [11], we have recently observed noticeable changes in their patterns of presentation, which are noteworthy of being thoroughly discussed.

 First, our experience mainly with Brazilian AIDS patients has recently demonstrated how prevalent PML could be as a neurological complication in HIV-1 seropositive patients, as PML cases are only exceeded by those of cerebral toxoplasmosis, cryptococcal meningoencephalitis, and CNS tuberculosis [12]. To date, the presentation of JCV-driven CNS manifestation is not restricted to multiple foci in the subcortical white matter [13], but also to multiple or unifocal lesions in a variety of sites, including the white matter of the cerebellar peduncles [8], the deep grey matter [14], neuronal cells in the cerebellum (granule cell neuronopathy) [3], or the cortical pyramidal neurons in the gray/white matter interface, recently termed as JCV encephalopathy [15]. These novel presentations of polyomavirus-associated diseases occur primarily in patients under HAART and highlight the relevance of performing biopsies and molecular studies to genotype and identify mutations altering the behavior and tropism of the JC virus in all HIV-1/AIDS patients with radiological observable brain lesions [13, 16, 17]. Noteworthy, the JCV-associated spectrum of neurological diseases currently constitutes a variety of syndromes recently classified as classic PML, inflammatory progressive multifocal leukoencephalopathy, JCV granule cell neuronopathy, and JCV meningitis [18]. As a result, we would like to propose a revision on the diagnostic criteria for PML as well as a complementary classification based on the number and location of the lesions (*multifocal* versus *unifocal*), and the nature of the clinical presentation—*classic* (subcortical white matter lesions) versus *variant* (involvement of the cerebellar peduncles and/or brainstem) (Figure 1(a)), which may be more suitable to what practitioners encounter in their everyday practice.

Furthermore, we have noticed a growing number of cases reporting worsening of PML after introduction of HAART [19–21]. This paradoxical worsening of PML weeks or months after starting HAART is due to an uncontrolled inflammatory response of the immune system to opportunistic pathogens and to certain tumors. The so-called “immune reconstitution syndrome” or “immune restoration disease” [4], when observed in the context of PML, can lead to rapid death in the absence of intervention; however, a consensus still needs to be reached about the proper measurements to take in this scenario (Figure 1(b)) [22].

Lastly, we would like to comment about the newly discovered neurotropism of BKV [23]. Although the recently characterized human polyomaviruses (KI, WU, WUV) have not yet been demonstrated to cause PML [24], there had been four cases of BKV-associated neurological compromise in AIDS patients reported in the literature; all of them accompanied by renal involvement and resulting in death [25–27]. Nevertheless, we have recently reported a case of BKV meningoencephalitis contained within the CNS, and with recovery under HAART [28]. Even when the causes of BKV neurotropism have not been established yet, we propose that this polyomavirus may be further investigated as an etiologic agent of neurological disease in AIDS patients, especially when more frequent etiologies have been discarded.

In conclusion, all the aforementioned data suggest that human polyomaviruses are playing a major role in the post-HAART era, and nothing but a multidisciplinary study of their biology and the pathogenesis of their associated diseases will lead to more efficacious interventions. 

## Figures and Tables

**Figure 1:**
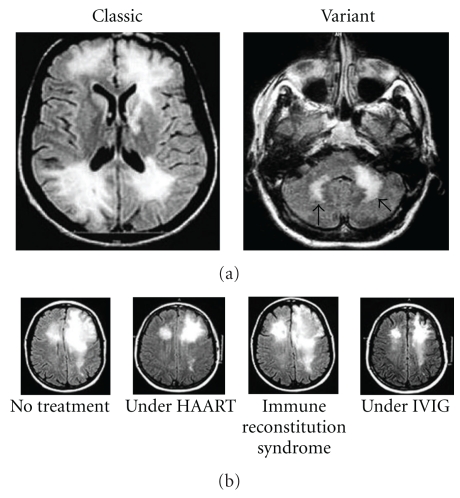
(a) Left, image shows classical PML, described as multiple foci of demyelination in the subcortical white matter. Right, image shows variant PML, described as one or multiple foci in regions outside the subcortical white matter, for example, the cerebellar peduncles (arrows). (b) Course and evolution of an AIDS patient with PML under HAART. From left to right, first column shows MRI with abnormal signal in the subcortical area of bilateral frontal lobes; diagnosis is confirmed by histopathology and treatment with HAART is started. The second column shows significant improvement under HAART. The third column shows worsening of the lesions three months after introduction of HAART (immune reconstitution syndrome). The fourth column shows improvement of the lesions after empiric treatment with intravenous immunoglobulin (IVIG).
